# In vitro activity of antibiotics potentially effective against difficult-to-treat strains of Gram-negative rods: retrospective study

**DOI:** 10.1038/s41598-024-59036-0

**Published:** 2024-04-09

**Authors:** Gabriela Kroneislová, Jan Závora, Vanda Gabriela Adámková, Anna Rýdlová, Václava Adámková

**Affiliations:** 1 Department of Clinical Microbiology and ATB Centre, Institute of Medical Biochemistry and Laboratory Diagnostic, First Faculty of Medicine, Charles University and General University Hospital, Ke Karlovu 2, Prague, 12808 Czech Republic; 2 Department of Biochemistry and Microbiology, Faculty of Food and Biochemical Technology, University of Chemistry and Technology, Prague, Czech Republic; 3 Department of Microbiology, Faculty of Medicine and Dentistry, Palacký University—Olomouc, Olomouc, Czech Republic; 4 Department of Plant Science, Cambridge University, Cambridge, UK; 5 Department of Infectious Disease, Faculty of Medicine, Imperial College London, London, UK

**Keywords:** Antimicrobial stewardship, Difficult-to-treat resistance, Aztreonam/avibactam, Cefiderocol, Ceftazidime/avibactam, Colistin, Ceftolozane/tazobactam, Fosfomycin, Imipenem/cilastatin/relebactam, Microbiology, Medical research

## Abstract

Bacterial resistance surveillance is one of the main outputs of microbiological laboratories and its results are important part of antimicrobial stewardship (AMS). In this study, the susceptibility of specific bacteria to selected antimicrobial agents was tested. The susceptibility of 90 unique isolates of pathogens of critical priority obtained from clinically valid samples of ICU patients in 2017–2021 was tested. 50% of these fulfilled difficult-to-treat resistance (DTR) criteria and 50% were susceptible to all antibiotics included in the definition. 10 Enterobacterales strains met DTR criteria, and 2 (20%) were resistant to colistin (COL), 2 (20%) to cefiderocol (FCR), 7 (70%) to imipenem/cilastatin/relebactam (I/R), 3 (30%) to ceftazidime/avibactam (CAT) and 5 (50%) to fosfomycin (FOS). For Enterobacterales we also tested aztreonam/avibactam (AZA) for which there are no breakpoints yet. The highest MIC of AZA observed was 1 mg/l, MIC range in the susceptible cohort was 0.032–0.064 mg/l and in the DTR cohort (incl. class B beta-lactamase producers) it was 0.064–1 mg/l. Two (13.3%) isolates of *Pseudomonas aeruginosa* (15 DTR strains) were resistant to COL, 1 (6.7%) to FCR, 13 (86.7%) to I/R, 5 (33.3%) to CAT, and 5 (33.3%) to ceftolozane/tazobactam. All isolates of *Acinetobacter baumannii* with DTR were susceptible to COL and FCR, and at the same time resistant to I/R and ampicillin/sulbactam. New antimicrobial agents are not 100% effective against DTR. Therefore, it is necessary to perform susceptibility testing of these antibiotics, use the data for surveillance (including local surveillance) and conform to AMS standards.

## Introduction

Since the discovery of the first antibiotic—penicillin—by Alexander Fleming in 1928, there have always been bacterial strains resistant to antibiotics. Already when accepting his Nobel Prize a few years later, Alexander Fleming mentioned increasing resistance as a possible problem^1^. Nowadays, antibiotic resistance is a global problem. Despite development of new antibiotics and improved infrastructure to access them, resistance to new agents is present even before they reach the market. According to ECDC (European Centre for Disease Prevention and Control), there are 670.000 cases of infections caused by resistant bacteria every year and 33.000 result in death^2^. It is predicted that the death rate will globally increase from 700.000 cases today to 10 million cases in 2050 because of antimicrobial-resistant pathogens^3^.

Infections caused by bacteria resistant to antibiotics is associated with failure of therapy, prolonged hospitalization, and increased cost of treatment. In order to address or possibly slow down the increasing resistance, it is important to introduce effective surveillance of not only hospital-acquired, but also community-acquired infections. This requires suitable classification of resistance phenotypes, so that the phenotypes can be easily and quickly sorted. The classification should also enable medical personnel to predict mortality and morbidity and thus have an impact on clinical practice.

The most commonly used classification nowadays was proposed by CDC and ECDC in 2008. This classification categorized resistance phenotypes according to the antibiogram of the strains: MDR (multi-drug resistance) describes nonsusceptibility to at least 1 antibiotic from at least 3 groups, XDR (extensive drug resistance) limits susceptibility to antibiotics from 2 or less groups and PDR (pan-drug resistance) represents nonsusceptible strains to all agents from all groups^4^.

Usage of these categories is widely accepted because they are easy-to-use and valuable for epidemiological purposes. However, their relevance is being recently disputed. MDR, XDR and PDR are defined solely by number of antibiotics/groups of antibiotics, but it does not comprehend which antibiotics are the strains resistant to. It is clinically important to know which antibiotics remain and can be used for therapy, and if they are more or less effective or toxic. Several studies have also shown that this phenotypic characterization does not correlate with mortality predictability^5^. Furthermore, it is necessary to determine resistance to wide range of antimicrobial agents for MDR/XDR/PDR classification, which is time consuming and not always applicable.

Kadri et al. made a proposal of a new antimicrobial resistance category for Gram-negative rods in 2018, and they called the category difficult-to-treat (DTR). This category includes bacterial strains resistant to all first-line antibiotics—in this case beta-lactams (penicillins, cephalosporins, carbapenems) and fluoroquinolones. Because of their high efficacy and relatively low toxicity, beta-lactams and fluoroquinolones are the most used antibiotics for Gram-negative infections^6^.

Treatment options for infections caused by strains resistant to first-line antibiotics are limited to less effective and more toxic antibiotics—such as aminoglycosides, tigecycline or colistin—and these are often associated with treatment failure and adverse effects^7–9^.

We conducted a retrospective study using bacterial strains isolated from clinically valid samples acquired from patients hospitalized in the ICUs of General University Hospital in Prague with approx. 1500 beds in 2017–2021. We determined the susceptibility of DTR strains isolated in this period to selected antibiotics with potential efficacy against DTR and compared it with the susceptibility of equal number of non-DTR strains. This work also emphasises the significance of DTR definition in communication of microbiologists and clinicians, which is important part of antimicrobial stewardship (AMS) and proper management of infections^10^.

## Methods

A retrospective monocentric study was conducted using 90 isolates of selected Gram-negative rods, 45 strains adhering to DTR definition and equal number of strains susceptible to all antibiotics included in it. Isolates of *Escherichia coli*, *Klebsiella pneumoniae*, *Enterobacter cloacae* complex, *Pseudomonas aeruginosa* and *Acinetobacter baumannii* were selected; they were obtained from clinically valid biological samples (blood cultures, abdominal fluids, lower respiratory tract samples) of patients hospitalized in the ICUs of a teaching hospital with approx. 1500 beds.

The susceptibility to antibiotics was tested by determining minimal inhibitory concentration (MIC); antibiotics for each pathogen were selected according to appropriateness in terms of therapy, see Table 1. Categories susceptible and resistant were established according to European Committee on Antimicrobial Susceptibility Testing (EUCAST) breakpoints if available^11^. Furthermore, we evaluated the effectiveness of tested antibiotics by analysing MIC distribution and calculating MIC_50_ and MIC_90_ (lowest concentration of an antimicrobial agent inhibiting 50% and 90% of isolates, respectively, in microbial population).

**Table 1:** Susceptibility to antibiotics tested.

	AMS	A/A	CAT	COL	C/T	FOS	FCR	I/R
Enterobacterales	−	+	+	+	−	+	+	+
*A. baumannii*	+	−	−	+	−	−	+	+
*P. aeruginosa*	−	+	+	+	+	+	+	+

“ + ” means tested, “−” means not tested.

*AMS* ampicillin/sulbactam, *A/A* aztreonam/avibactam, *CAT* ceftazidime/avibactam, *COL* colistin, *C/T* ceftolozane/tazobactam, *FOS* fosfomycin, *FCR* cefiderocol, *I/R* imipenem/cilastatin/relebactam.

### Susceptibility testing

Broth microdilution method was used for determination of colistin (COL) susceptibility, agar dilution for fosfomycin (FOS) and the method of gradient strips for the rest of the antibiotics: ampicillin/sulbactam (AMS), aztreonam/avibactam (A/A), cefiderocol (FCR), ceftazidime/avibactam (CAT), ceftolozane/tazobactam (C/T) and imipenem/relebactam (I/R).

Control strains *Escherichia coli* ATCC 25,922 and ATCC 35,218, *Pseudomonas aeruginosa* ATCC 27,853, Klebsiella pneumoniae ATCC 700,603 and ATCC BAA-2814 were used for quality control purposes according to EUCAST.

### Broth microdilution

The suspensions of bacterial strains cultured overnight on Columbia blood agar (OXOID/Thermo Scientific, Waltham, Massachusetts, USA) adjusted to 0.5 McFarland turbidity were inoculated into the wells of MIKROLATEST® plates (Erba Lachema, Brno, Czech Republic) according to the test instructions and it was incubated at 37 °C for 24 h. The MIC values were recorded as the lowest concentrations of COL inhibiting visible growth completely.

### Agar dilution

Agar dilution was performed using a commercial kit AD Fosfomycin 0,25–256 (Liofilchem, Roseto degli Abruzzi, Italy). The suspensions adjusted to 0.5 McF were diluted 10 times in 0.9% NaCl solution and 2 µl of the final suspension was spotted into the wells of the agar dilution plates, which were incubated at 37 °C for 24 h. The MIC values were recorded as the lowest concentrations of FOS inhibiting growth completely.

### Gradient strips

The gradient strip method for C/T and CAT was performed using Etest® (bioMérieux SA, Marcy-l’Etoile, France), and for FCR, I/R, A/A, AMS MIC Test Strip (MTS, Liofilchem, Roseto degli Abruzzi, Italy) was used. The bacterial suspensions were adjusted to 0.5 McF turbidity. They were then inoculated on Mueller Hinton agar for Etest® (MHE agar; bioMérieux SA, Marcy l’Etoile, France), and the strips were applied on the plates and incubated in 37 °C for 24 h.

### Note on interpretation of the results

There are breakpoints available for interpretation only in some of the antibiotics tested and only for some bacteria. If there were breakpoints available, the result of susceptibility testing was determined. If the breakpoints were not published, only the distribution of MICs and MIC_50_/MIC_90_ values were analysed, but the percentage of resistance was not calculated.

## Results

**Figure 1:**
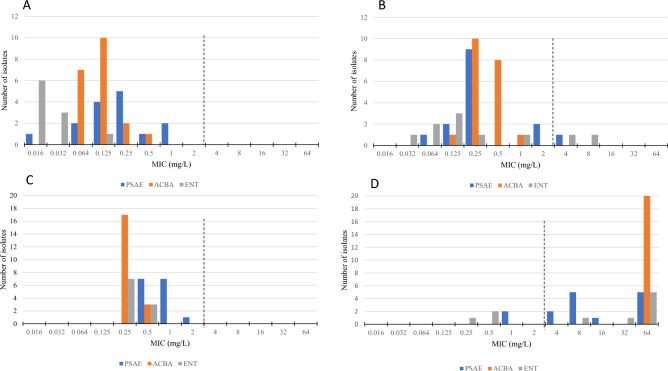
MIC (minimum inhibitory concentration) distributions; A, Cefiderocol non-DTR; B, Cefiderocol DTR; C, Imipenem/cilastatin/relebactam non-DTR; D, Imipenem/cilastatin/relebactam DTR. PSAE, *Pseudomonas aeruginosa*; ACBA, *Acinetobacter baumannii*; ENT, Enterobacterales. Dotted lines represent EUCAST 2023 clinical breakpoints of cefiderocol (PSAE, ENT susceptible, MIC ≤ 2 mg/L, ACBA has no clinical breakpoints), imipenem/cilastatin/relebactam (PSAE, ACBA, ENT susceptible, MIC ≤ 2 mg/L).

From 2017 to 2021, 2594 episodes of severe infections caused by selected Gram-negative rods (*E. coli*, *K. pneumoniae*, *E. cloacae* complex, *P. aeruginosa*, *A. baumannii*) were reported in our setting; 4.9% (n = 127) of these were associated with DTR phenotype. The prevalence of DTR was the highest in *A. baumannii* (46.6%) and *P. aeruginosa* (11.4%). DTR is relatively rare in Enterobacterales with only 22 strains (1.2%). The results of susceptibility testing and MIC_50_ and MIC_90_ compared in both groups and calculated from total number of strains are shown in Table 2 and Fig. 1.

**Table 2:** MIC distribution and percentage of resistance to antibiotics.

		*Acinetobacter baumannii*			Enterobacterales			*Pseudomonas aeruginosa*		
		S (n = 20)	DTR (n = 20)	total (n = 40)	S (n = 10)	DTR (n = 10)	total (n = 20)	S (n = 15)	DTR (n = 15)	total (n = 30)
AMS	MIC_50_ (mg/l)	0.016	> 256	1	NT	NT	NT	NT	NT	NT
	MIC_90_ (mg/l)	0.016	> 256	> 256	NT	NT	NT	NT	NT	NT
	range (mg/l)	0.016 to 1	> 256	0.016 to > 256	NT	NT	NT	NT	NT	NT
	% resistant (n)				NT	NT	NT	NT	NT	NT
A/A	MIC_50_ (mg/l)	NT	NT	NT	0.032	0.25	0.064	4	16	4
	MIC_90_ (mg/l)	NT	NT	NT	0.064	0.5	0.5	4	32	32
	range (mg/l)	NT	NT	NT	0.032 to 0.064	0.064 to 1	0.032 to 1	2 to 8	4 to 64	2 to 64
	% resistant (n)	NT	NT	NT						
FCR	MIC_50_ (mg/l)	0.125	0.25	0.25	0.016	0.125	0.032	0.25	0.25	0.25
	MIC_90_ (mg/l)	0.25	0.5	0.5	0.032	4	1	1	2	1
	range (mg/l)	0.064 to 0.5	0.125 to 1	0.064 to 1	0.016 to 0.125	0.032 to 8	0.016 to 8	0.016 to 1	0.064 to 4	0.016 to 4
	% resistant (n)				0 (0)	20 (2)	10 (2)	0 (0)	6.7 (1)	3.3 (1)
CAT	MIC_50_ (mg/l)	NT	NT	NT	0.125	2	0.25	1	8	4
	MIC_90_ (mg/l)	NT	NT	NT	0.25	> 256	16	2	> 256	32
	range (mg/l)	NT	NT	NT	0.125 to 0.25	0.25 to > 256	0.125 to > 256	0.5 to 4	4 to > 256	0.5 to > 256
	% resistant (n)	NT	NT	NT	0 (0)	30 (3)	15 (3)	0 (0)	33.3 (5)	16.7 (5)
CTL	MIC_50_ (mg/l)	NT	NT	NT	NT	NT	NT	0.5	2	1
	MIC_90_ (mg/l)	NT	NT	NT	NT	NT	NT	1	> 256	32
	range (mg/l)	NT	NT	NT	NT	NT	NT	0.25 to 1	1 to > 256	0.25 to > 256
	% resistant (n)	NT	NT	NT	NT	NT	NT	0 (0)	33.3 (5)	16.7 (5)
COL	MIC_50_ (mg/l)	1	1	1	0.5	0.5	0.5	2	1	2
	MIC_90_ (mg/l)	1	1	1	1	8	8	8	> 16	16
	range (mg/l)	0.5 to 2	0.5 to 1	0.5 to 2	0.25 to > 16	0.5 to 16	0.25 to > 16	1 to 16	0.5 to > 16	0.5 to > 16
	% resistant (n)	0 (0)	0 (0)	0 (0)	10 (1)	20 (2)	15 (3)	20 (3)	13.3 (2)	16.7 (5)
FOS	MIC_50_ (mg/l)	NT	NT	NT	16	32	16	64	32	32
	MIC_90_ (mg/l)	NT	NT	NT	32	128	128	128	> 256	> 256
	range (mg/l)	NT	NT	NT	0.5 to 64	8 to 256	0.5 to 256	4 to > 256	4 to > 256	4 to > 256
	% resistant (n)	NT	NT	NT	10 (1)	50 (5)	30 (6)			
I/R	MIC_50_ (mg/l)	0.25	64	0.5	0.25	32	0.5	1	8	1
	MIC_90_ (mg/l)	0.5	64	64	0.5	64	64	1	64	64
	range (mg/l)	0.25 to 0.5	64	0.25 to 64	0.25 to 0.5	0.25 to 64	0.25 to 64	0.5 to 2	1 to 64	0.5 to 64
	% resistant (n)	0 (0)	100 (20)	50 (20)	0 (0)	70 (7)	35 (7)	0 (0)	86.7 (13)	43.3 (13)

*AMS* ampicillin/sulbactam; *A/A* aztreonam/avibactam; *FCR* cefiderocol; *CAT* ceftazidime/avibactam; *COL* colistin; *CTL* ceftolozane/tazobactam; *DTR* difficult-to-treat; *FOS* fosfomycin; *I/R* imipenem/cilastatin/relebactam; *MIC* minimum inhibitory concentration; *NT* not tested; *S* susceptible isolates.

Imipenem/cilastatin/relebactam, ceftazidime/avibactam and ceftolozane/tazobactam were tested with a fixed concentration of 4 mg/l of relebactam, avibactam and tazobactam.

Empty fields illustrate nonavailability of clinical breakpoints.

### A. baumannii

For *A. baumannii*, the resistance to COL is not dependent on DTR/non-DTR status of the strain as all strains were susceptible to COL. Moreover, the distribution of MICs was identical when comparing both groups. It is not possible to interpret the results of FCR susceptibility testing, but the MICs were relatively low (range 0.064 to 1 mg/l in total), and the distribution was similar in both groups. 100% *A. baumannii* strains from non-DTR group were susceptible to AMS and I/R and 100% DTR strains were resistant to these antibiotics.

### P. aeruginosa

**Figure 2:**
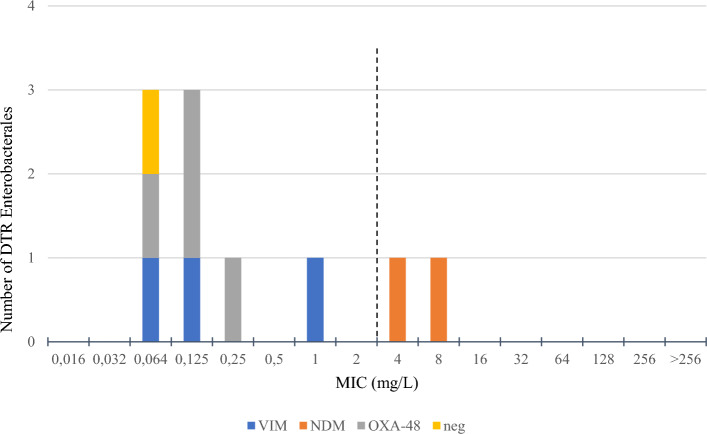
MIC of cefiderocol according to mechanism of resistance. Dotted line represents EUCAST 2023 clinical breakpoint of cefiderocol (susceptible, MIC ≤ 2 mg/L).

Testing the susceptibility of *P. aeruginosa* to COL revealed that the distribution of MICs (MIC range was 1 to 16 in non-DTR and 1 to > 16 in DTR) and percentage of resistance (20% and 13.3%, resp.) is comparable between both groups. Results of cefiderocol susceptibility testing showed similar results in both groups (see Fig. 2). 86.7% DTR strains were resistant to I/R compared to 100% susceptible in non-DTR group. Both CAT and C/T showed the same percentage of resistance: 0% isolates resistant isolates were found in non-DTR group and 33.3% resistant in DTR group. However, the distribution of MICs was shifting slightly in favour of C/T: MIC_50_ is 2 mg/l for DTR in C/T and 8 mg/l in CAT.

### Enterobacterales

The resistance of Enterobacterales to COL was 10% in non-DTR and 20% in DTR. There were only 2 DTR strains (20%) resistant to FCR while 100% non-DTR strains were susceptible. Mechanisms of resistance to beta-lactams in Enterobacterales were analysed and both strains resistant to FCR were producers of carbapenemase type NDM (see Fig. 2). Non-DTR Enterobacterales were all susceptible to CAT with 3 DTR strains (30%) found to be resistant.

## Discussion

This retrospective study analysed and compared the susceptibility patterns of bacterial isolates defined as difficult-to-treat (DTR) and non-DTR. The importance of DTR classification and its superiority over conventional phenotype classifications (MDR, XDR, PDR) lies in the DTR classification emphasis on the class of the resistant antibiotic. As it specifies which agent is resistant, this classification becomes more applicable for clinical practice, and makes the communication between clinicians and microbiologists more straightforward. Studies have also shown that the DTR status was better predictor of mortality than the conventional classifications^4,6,12^.

Gram-negative bacteria are causative agents of severe infections, incl. sepsis. According to EPIC III study including 15,000 ICU patients from 88 countries, 67% of positive blood cultures in patients with bloodstream infection episodes contained Gram-negative pathogens^13^. If these pathogens are antimicrobial-resistant, it adds difficulties with treatment. In these cases, it is particularly important to determine susceptibility of the strains causing the infection, so proper antibiotic therapy can be prescribed in time^14,15^.

DTR definition includes Gram-negative bacteria ranked as critical priority pathogens by the World Health Organization (WHO): carbapenem-resistant *A. baumannii*, carbapenem-resistant *P. aeruginosa*, carbapenem-resistant, and third-generation cephalosporin-resistant Enterobacterales^16^. However, not all these pathogens are DTR. It was previously described that resistance to carbapenems in *A. baumannii* was a good predicting factor of DTR phenotype. But, especially with *P. aeruginosa*, there are strains with retained susceptibility to other beta-lactams or fluoroquinolones, therefore not meeting DTR definition^12,17^.

In the setting of this study, the prevalence of DTR Gram-negatives in clinically valid samples was higher than what has reported Kadri et al. in their study on samples from bloodstream infection (BSI) episodes: 1% of DTR overall and *A. baumannii* 18.3%, *P. aeruginosa* 2.3% and Enterobacterales 0.46%. This difference can be due to the source of the isolates (BSI vs. various severe infections)^6^.

If infection caused by a DTR strain occurs, the therapy requires antibiotics usually more toxic and less effective than the first choice (e.g., colistin, aminoglycosides, tigecycline). Colistin is notoriously problematic as both EUCAST and Clinical and Laboratory Standards Institute (CLSI) are expressing limitations about testing and prescribing colistin. Until recently, colistin was routinely prescribed as a “last resort” antibiotic when treating difficult-to-treat infections. A document on colistin susceptibility testing published by EUCAST in 2021 states that colistin breakpoints should not be used for clinical interpretation but only for differentiation of strains with acquired resistance to colistin. Furthermore, according to the document, it is not advised to prescribe colistin in monotherapy (excl. noncomplicated urinary tract infections). CLSI categorizes all isolates of Gram-negative bacteria as nonsusceptible to colistin because PK/PD data show poor clinical efficacy^11,17^. There are also problems with susceptibility testing of colistin. It is recommended to perform broth microdilution because of poor diffusion of colistin in agar, suggesting that all agar methods are inadequate^18^. In this study, the resistance to colistin was not dependent on DTR/non-DTR status of the strain, as was observed by other authors^19,20^.

Another older antibiotic with a potential effect against resistant pathogens is fosfomycin. It was recently rehabilitated, and its importance is widely considered. Several studies have shown broad spectrum of activity against Gram negatives. However, similarly to colistin, there were difficulties with determining susceptibility to this agent. Gold standard for testing is agar dilution, which is a time-consuming method unsuitable for routine practice. It remains to be seen, which other method could be appropriate^21,22^. The results of fosfomycin susceptibility testing in this study were analogous to colistin: there was no difference in MIC distribution between non-DTR and DTR isolates. MIC values were close to the breakpoint in both groups, which can predict future resistance even in non-DTR category.

After DTR definition was established, new antibiotics or new combinations of antibiotics with beta-lactamase inhibitors were developed.

Ceftazidime/avibactam is a combination of established third-generation cephalosporin with a new non-beta-lactam beta-lactamase inhibitor. It was approved for treatment of complicated urinary tract infections (cUTI), complicated intraabdominal infections (cIAI), hospital-acquired pneumonia (HAP) and ventilator-acquired pneumonia (VAP) and for infections due to aerobic Gram-negative rods in patients with limited treatment options. This combination is effective against beta-lactamases from Ambler class A, C, and some from class D, which was reflected in the results of this study—all Enterobacterales strains resistant to ceftazidime/avibactam were producing metallo-beta-lactamases (MBL, class B beta-lactamases). Especially in patients with KPC-producing *Klebsiella pneumoniae* infections, ceftazidime/avibactam was deemed to be a good alternative to colistin, particularly as the risk of nephrotoxicity was notably lower. ^23^ Infections caused by *P. aeruginosa* strains producing class A carbapenemases (e.g., GES) may require treatment with ceftazidime/avibactam, because of resistance to ceftolozane/tazobactam^24,25^.

Ceftolozane/tazobactam is a combination of novel fifth-generation cephalosporin and older beta-lactam beta-lactamase inhibitor and its uses include cIAI (with metronidazole), cUTI, HAP and VAP. Ceftolozane was originally developed as an antipseudomonal agent. According to several studies, its effect against *P. aeruginosa* (including resistant strains) was better than ceftazidime/avibactam with lower MICs of ceftolozane/tazobactam. On the other hand, the effect against Enterobacterales was better with ceftazidime/avibactam^26^. In this cohort, the results were similar as MICs of ceftolozane/tazobactam were slightly lower than ceftazidime/avibactam in *P. aeruginosa* strains. The effect of ceftolozane was shown to be superior against *P. aeruginosa* excluding strains producing MBL^27^.

Imipenem/cilastatin/relebactam combines older carbapenem and novel non-beta-lactam beta-lactamase inhibitor, it is approved for cUTI, cIAI, HAP and VAP in adults. Because of the addition of relebactam, the effect of imipenem is broadened to class A and C beta-lactamase producers, however it is not active against MBL and type OXA carbapenemases. Results of this study are concordant with other works. There were distinct differences between DTR and non-DTR groups *A. baumannii* with all DTR being resistant due to OXA carbapenemase production and all non-DTR were susceptible. There are several susceptible strains of DTR *P. aeruginosa* and Enterobacterales, including producers of class A carbapenemases and carbapenemase non-producers^24,25,27,28^.

The revival of aztreonam by combining it with avibactam shows potential against KPC, MBL and class C carbapenemases. There are no interpretation criteria for aztreonam/avibactam, but there were differences between non-DTR and DTR groups, MICs were significantly lower in Enterobacterales compared to *P. aeruginosa*. It was reported that the effect on carbapenem-resistant *P. aeruginosa* was superior when using ceftazidime/avibactam when compared to aztreonam/avibactam^29,30^.

A novel siderophore cephalosporin cefiderocol uses transport mechanism for iron and thus penetrates bacterial cell wall. This way, it overcomes all classes of beta-lactamases and other mechanisms of resistance (porin channel mutations, efflux pump overexpression). Cefiderocol showed high activity against DTR isolates of Enterobacterales, *P. aeruginosa* and *A. baumannii*, which was also reported in this study. Cefiderocol is one of the most promising agents against DTR, but there are problems with availability (i.e., it was not on the market in the Czech Republic when this manuscript was finalized)^31–34^.

The main limitation of this study is that it was a single centre study. The cohort diversity was dependent on the distribution of hospitalized patients of one teaching hospital, and therefore it may not reflect the epidemiological situation of the rest of the Czech Republic. Single-centre nature of this study is, however, also beneficial. In some cases, multicentric studies are based on data provided to the authors and authors cannot inspect them further. There are international surveillance systems (e.g., EARS-Net) collecting these data, but some regions are represented only by one facility, which could add bias.

## Conclusions

Prompt and correct classification of resistance phenotypes is one of the tools of antimicrobial stewardship (AMS). DTR phenotype is a great challenge for AMS, especially on regional level. According to the results of this study, it still important to determine susceptibility using phenotypic methods and it is apparent that detection of resistance mechanism by genotypic methods alone is not enough for proper antibiotic treatment. Because phenotypic methods can be time-consuming, it is crucial for the laboratories to maintain the shortest time-to-result as possible.

There are several new agents available with the potential to treat infections caused by DTR isolates. In vitro activity of these agents can be determined by laboratory methods, but there are no interpretation criteria for some of them. Clinical application of in vitro testing can be limited because of nonavailability on the market, unknown adverse effects, and interactions of new antimicrobial agents.

## Data Availability

All data can be accessed via the corresponding author.

## References

[1] Rosenblatt-Farrell N (2009). The landscape of antibiotic resistance. Environ. Health Perspect..

[2] WHO Regional Office for Europe/European Centre for Disease Prevention and Control. Antimicrobial resistance surveillance in Europe 2022–2020 data. Copenhagen: WHO Regional Office for Europe; 2022.

[3] Review on Antimicrobial Resistance. Antimicrobial resistance: Tackling a crisis for the health and wealth of nations. 2014. https://amr-review.org/sites/default/files/AMR%20Review%20Paper%20-%20Tackling%20a%20crisis%20for%20the%20health%20and%20wealth%20of%20nations_1.pdf [Accessed 7.3.2023].

[4] Magiorakos AP, Srinivasan A, Carey RB (2012). Multidrug-resistant, extensively drug-resistant and pandrug-resistant bacteria: an international expert proposal for interim standard definitions for acquired resistance. Clin. Microbiol. Infect..

[5] Vardakas KZ, Rafailidis PI, Konstantelias AA, Falagas ME (2013). Predictors of mortality in patients with infections due to multi-drug resistant Gram negative bacteria: the study, the patient, the bug or the drug?. J. Infect..

[6] Kadri SS, Adjemian J, Lai YL (2018). Difficult-to-treat resistance in gram-negative bacteremia at 173 US hospitals: Retrospective cohort analysis of prevalence, predictors, and outcome of resistance to all first-line agents. Clin. Infect. Dis..

[7] Drawz SM, Bonomo RA (2010). Three decades of beta-lactamase inhibitors. Clin. Microbiol. Rev..

[8] Bush K, Jacoby GA (2010). Updated functional classification of beta-lactamases. Antimicrob. Agents Chemother..

[9] Cisneros JM, Rosso-Fernández CM, Roca-Oporto C (2019). Colistin versus meropenem in the empirical treatment of ventilator-associated pneumonia (Magic Bullet study): An investigator-driven, open-label, randomized, noninferiority controlled trial. Crit. Care.

[10] Kroneislova G, Zavora J, Adamkova V (2023). Are new antibiotics efficient against DTR (difficult-to-treat resistance) isolates? Prevalence and susceptibility of invasive DTR strains. Crit. Care.

[11] The European Committee on Antimicrobial Susceptibility Testing. Breakpoint tables for interpretation of MICs and zone diameters. 2023., Version 13.0. http://www.eucast.org. [Accessed 7. 3. 2023].

[12] Huh K, Chung DR, Ha YE (2020). Impact of difficult-to-treat resistance in gram-negative bacteremia on mortality: Retrospective analysis of nationwide surveillance data. Clin Infect Dis..

[13] Vincent JL, Sakr Y, Singer M (2020). Prevalence and outcomes of infection among patients in intensive care units in 2017. JAMA..

[14] Niederman MS, Baron RM, Bouadma L (2021). Initial antimicrobial management of sepsis. Crit. Care.

[15] Falcone M, Bassetti M, Tiseo G (2020). Time to appropriate antibiotic therapy is a predictor of outcome in patients with bloodstream infection caused by KPC-producing *Klebsiella pneumoniae*. Crit. Care.

[16] Kanj SS, Bassetti M, Kiratisin P (2022). Clinical data from studies involving novel antibiotics to treat multidrug-resistant Gram-negative bacterial infections. Int. J. Antimicrob. Agents.

[17] Clinical and Laboratory Standards Institute. Performance standards for antimicrobial susceptibility testing; 33rd edition. www.clsi.org [Accessed 7. 3. 2023].

[18] Matuschek E, Åhman J, Webster C, Kahlmeter G (2018). Antimicrobial susceptibility testing of colistin: Evaluation of seven commercial MIC products against standard broth microdilution for *Escherichia coli*, *Klebsiella pneumoniae*, *Pseudomonas aeruginosa*, and Acinetobacter spp. Clin. Microbiol. Infect..

[19] Adámková V, Mareković I, Szabó J (2022). Antimicrobial activity of ceftazidime-avibactam and comparators against Pseudomonas aeruginosa and Enterobacterales collected in Croatia, Czech Republic, Hungary, Poland, Latvia and Lithuania: ATLAS Surveillance Program, 2019. Eur. J. Clin. Microbiol. Infect. Dis..

[20] Pruss A, Kwiatkowski P, Masiuk H (2022). Analysis of the prevalence of colistin resistance among clinical strains of *Klebsiella pneumoniae*. Ann. Agric. Environ. Med..

[21] Smith EC, Brigman HV, Anderson JC (2020). Performance of four fosfomycin susceptibility testing methods against an international collection of clinical *Pseudomonas aeruginosa* isolates. J. Clin. Microbiol..

[22] Behera B, Mohanty S, Sahu S, Praharaj AK (2018). In vitro activity of fosfomycin against multidrug-resistant urinary and nonurinary gram-negative isolates. Indian J. Crit. Care Med..

[23] van Duin D, Lok JJ, Earley M (2018). Colistin versus ceftazidime–avibactam in the treatment of infections due to carbapenem–resistant enterobacteriaceae. Clin. Infect. Dis..

[24] Bassetti M, Vena A, Sepulcri C, Giacobbe DR, Peghin M (2020). Treatment of bloodstream infections due to gram-negative bacteria with difficult-to-treat resistance. Antibiotics.

[25] Karlowsky JA, Lob SH, DeRyke CA (2022). In vitro activity of ceftolozane–tazobactam, imipenem–relebactam, ceftazidime–avibactam, and comparators against *Pseudomonas aeruginosa* isolates collected in United States hospitals according to results from the SMART surveillance program, 2018 to 2020. Antimicrob. Agents Chemother..

[26] Alatoom A, Elsayed H, Lawlor K (2017). Comparison of antimicrobial activity between ceftolozane–tazobactam and ceftazidime–avibactam against multidrug-resistant isolates of *Escherichia coli*, *Klebsiella pneumoniae*, and *Pseudomonas aeruginosa*. Int. J. Infect. Dis..

[27] Lob SH, DePestel DD, DeRyke CA (2021). Ceftolozane/tazobactam and imipenem/relebactam cross-susceptibility among clinical isolates of *Pseudomonas aeruginosa* from patients with respiratory tract infections in ICU and Non-ICU wards-SMART United States 2017–2019. Open Forum Infect. Dis..

[28] Hernández-García M, García-Castillo M, Melo-Cristino J (2022). In vitro activity of imipenem/relebactam against Pseudomonas aeruginosa isolates recovered from ICU patients in Spain and Portugal (SUPERIOR and STEP studies). J. Antimicrob. Chemother..

[29] Yu W, Xiong L, Luo Q (2021). In vitro activity comparison of ceftazidime–avibactam and aztreonam–avibactam against bloodstream infections with carbapenem-resistant organisms in China. Front. Cell Infect. Microbiol..

[30] Rossolini GM, Stone G, Kantecki M, Arhin FF (2022). In vitro activity of aztreonam/avibactam against isolates of Enterobacterales collected globally from ATLAS in 2019. J. Glob. Antimicrob. Resist..

[31] Hoellinger B, Simand C, Jeannot K (2023). Real-world clinical outcome of cefiderocol for treatment of multidrug-resistant non-fermenting, gram negative bacilli infections: a case series. Clin. Microbiol. Infect..

[32] Candel FJ, Santerre Henriksen A, Longshaw C, Yamano Y, Oliver A (2022). In vitro activity of the novel siderophore cephalosporin, cefiderocol, in Gram-negative pathogens in Europe by site of infection. Clin. Microbiol. Infect..

[33] Pascale R, Pasquini Z, Bartoletti M (2021). Cefiderocol treatment for carbapenem-resistant *Acinetobacter baumannii* infection in the ICU during the COVID-19 pandemic: a multicentre cohort study. JAC Antimicrob. Resist..

[34] Paul M, Carrara E, Retamar P (2022). European Society of Clinical Microbiology and Infectious Diseases (ESCMID) guidelines for the treatment of infections caused by multidrug-resistant Gram-negative bacilli (endorsed by European society of intensive care medicine). Clin. Microbiol. Infect..

